# Thiol-free oligonucleotide surface modification of gold nanoparticles for nanostructure assembly†

**DOI:** 10.1039/c8na00148k

**Published:** 2018-09-20

**Authors:** Anastasia O. Maslova, I. Ming Hsing

**Affiliations:** Department of Chemical and Biological Engineering, The Hong Kong University of Science and Technology Hong Kong China kehsing@ust.hk; Bioengineering Graduate Program, The Hong Kong University of Science and Technology Hong Kong China

## Abstract

Gold nanoparticles (AuNPs) decorated with thiol-modified DNA (HS-DNA) strands are an extensively studied, easily adjustable, and highly controllable material for constructing 3D nanostructures with various shapes and functions. However, few reproducible and robust methods involving DNA templates as a key reagent are available for obtaining 3D nanoparticle assemblies. It is still challenging to strictly control the number and location of DNA strands on the AuNP surface. Here, we introduce an efficient approach for the surface modification of AuNPs using unmodified DNA oligonucleotides by building DNA cages that trap the nanoparticles. This enables us to vary the process of nanostructure assembly and create anisotropic nanoparticles that are necessary for directed structure construction. This developed method simplifies the production process in comparison with conventional HS-DNA modification protocols and helps to precisely control the density and position of functional DNA strands designed for further hybridization with other AuNP conjugates.

## Abstract 2

Thiol-free DNA scaffold assembly method has been developed for AuNPs surface modification that allows to control the position of functional strands for further nucleic acid circuit.

## Graphical abstract

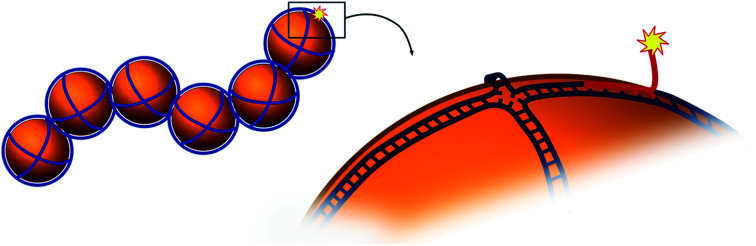

## Introduction

Nanoscale materials have attracted a lot of attention due to their chemical and physical properties, and can be used in the detection of small molecules and the miniaturization of optical and biomedical devices. The controlled interaction of gold nanoparticles has been widely studied for its potential applications in genomics, laser phototherapy, sensing, drug delivery, and optical and energy fields.^1–5^ DNA is also used for the surface modification of nanoparticles in order to provide a robust and programmable starting material when creating self-assembled nanostructures.^6,7^ This is useful in constructing a framework for nanoparticle arrays of different dimensions.

Surface modification of gold nanoparticles is often required for the assembly of 1D/2D/3D nanoparticles. However, the process of synthesizing asymmetrically functionalized AuNPs is complex and the results, although reproducible, suffer from side products.^8^ The first method for surface modification was introduced independently by Mirkin *et al.*^9^ and Alivisatos *et al.*^10^ They developed approaches providing high-density DNA coverage of the nanoparticle surface and low-density coverage, respectively. Their method utilizes thiol chemistry, and an excess of single-stranded DNA (ssDNA) oligonucleotides is necessary for attaching the strands to the gold. ssDNA strands allow the aggregation of nanoparticles through the hybridization process when the target complimentary strand is introduced into solution. Although this method is highly reproducible, one might want to control the stoichiometry and the placement of the DNA strands, and reduce the number of oligonucleotides on the surface to achieve directed, and not random, structure growth and assembly. Several methods were introduced over the years, including dATP-assisted surface modification,^11,12^ and a modified^13^ Taton's protocol^14^ for low-density surface functionalization, but they still do not enable full control over the assembly of the nanoparticles. Furthermore, it is impossible to tailor-make multiple DNA strands in specific positions on a nanoparticle's surface based only on the optimized concentration ratios between thiol ssDNAs and AuNPs. The most that can be achieved is the attachment of several DNA strands in stochastic positions. To guide the assembly process, several methods were developed. Sleiman's group^13^ suggested the use of assisting DNA cages to place desired thiolated oligonucleotides in a designated pattern on the surface of the AuNP. This approach requires many steps, including washing, purification, and denaturation procedures, and is still limited to a template shape that directs the placement of functional DNA strands on the AuNP. Another low density approach introduced by Pei *et al.*^15^ where the number of functional ssDNA strands was controlled by polyA tails that served as an anchoring sequence without using thiol modification of DNA. Schreiber *et al.*^16^ came up with the idea of using DNA origami as a protective shell from high salt-induced aggregation and as an assistive structure for further nanostructure assembly. However, as in most origami procedures, this method requires a large amount of DNA.

Although DNA is a very good material for facilitating the formation of nanostructures from AuNPs at a nanoscale level, it remains challenging to grow nanostructures from 1D to 2D and 3D patterns.^17^ Encapsulating a nanoparticle in a DNA cage is an interesting approach. There are numerous reports on building DNA cages for cargo delivery that demonstrate the robustness and reproducibility of this method. AuNPs are known to be a good support material for DNA polyhedrons.^18–21^ Origami cages^22^ can accommodate nanoparticles inside, restrict the surface of nanoparticles, and form asymmetric hybridization sites for growing complexes.

Our approach uses unmodified DNA oligonucleotides to create anisotropic AuNPs with the further generation of nanostructures without using DNA origami-based structures as a lattice that supports the AuNPs. DNA oligonucleotides form a cage that traps the AuNP, can easily adapt to the form of the nanoparticle, and does not involve large amounts of DNA. The cage is octahedral in shape, with each edge consisting of double-stranded DNA (dsDNA) with ssDNA regions, which are used to help control the placement of reactant oligonucleotides. The construction of nanostructures depends on the hybridization process between hanging complementary regions of the DNA cage.

Functionalized DNA conjugates can then be used as building blocks for electronic devices based on linear chains and self-assembled monolayers (SAMs),^23–25^ where AuNPs bring electrical function to a non-conductive DNA scaffold. The advantage of this method over similar approaches is that the assembly process is consistent in design simplicity and in the reduced use of DNA in the supporting scaffold. Controlled nanoparticle-based nanostructures using nucleic acid circuits and functionalized AuNPs are likely to have useful applications in diagnostic and therapeutic fields.

## Materials and methods

### Materials

Gold nanoparticles (diameter 10 nm, stabilized with a citrate buffer) were purchased from Sigma-Aldrich. All oligonucleotides were purchased from Integrated DNA Technologies (IDT) with PAGE purification. All buffers were prepared according to common protocols using Sigma production, and were dissolved in Milli-Q pure water.

### Preparation of DNA–AuNP complexes

#### dATP protocol

Since citrate-stabilized AuNPs aggregate in solutions that contain Mg^2+^ ions, the AuNPs were coated with *O*-(2-carboxyethyl)-*O*′-(2-mercaptoethyl)heptaethylene glycol (thiol-OEG) according to the protocol developed earlier.^11,12^ Briefly, citrate-stabilized AuNPs were first incubated with dATP in the molar ratio dATP/AuNPs = 1000/1 for 15 minutes. Then the mixture was added to a sodium phosphate buffered saline solution (PBS: 10 mM Na_2_HPO_4_/NaH_2_PO_4_, pH 8.0 and 0.1 M NaCl). After light vortexing, 1 mM thiol-OEG was introduced in the molar ratio OEG/AuNPs = 1000/1, followed by heating at 60 °C for three hours. Then, AuNPs were washed three times in 10 mM PBS (pH 8.0) with 0.1 M NaCl through centrifugation (15 000 rpm for 35 minutes at 4 °C) to remove excess reagents. The obtained conjugates were resuspended in 100 μL 10 mM PBS (pH 8.0) with 0.3 M NaCl.

#### Octahedron conjugation

**Scheme 1:**
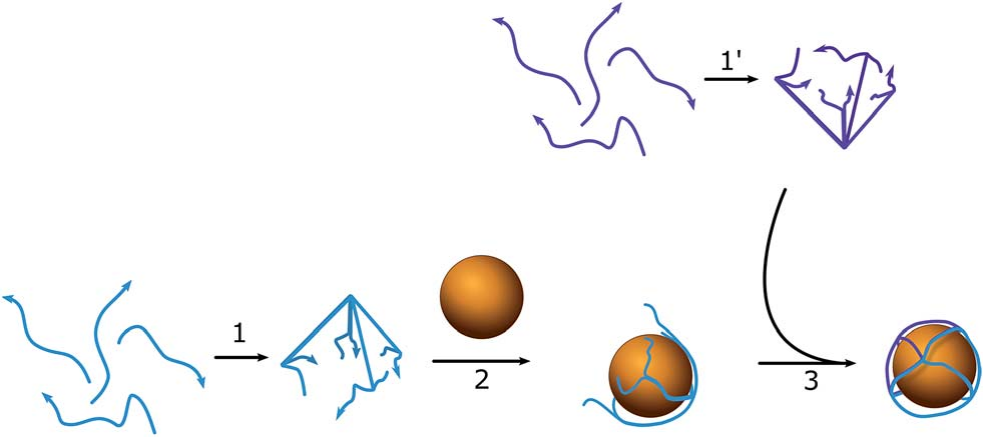
Schematic of the assembly process for a DNA–AuNP conjugate. (1) and (1′) are the preassembly processes of hemispheres A and B; (2) mixture of AuNPs with the first batch of hemispheres (A); (3) addition of the second hemisphere (B) and formation of the full structure.

An octahedronal cage was assembled in two steps (Scheme 1): two semi-spheres were built first, and then they were mixed with AuNPs to make the complete structure. An octahedronal semi-sphere was assembled as follows: a mixture of four DNA oligonucleotides (Table S1†) with a final concentration of 4 μM in 10 mM PBS for each strand was heated to 95 °C and slowly cooled down to room temperature over four hours. Then, 50 μL of 50 nM AuNPs were incubated with two types of semi-spheres (1 μL each) at room temperature overnight. After that, the AuNPs were purified by centrifuging at 15 000 rpm and 4 °C for 30 minutes and washed with 10 mM PBS with 0.1 M NaCl. The presence of DNA at the surface was demonstrated using 1% agarose gel electrophoresis (8 V cm^−1^, 1 h, 0.5× TBE), and the effective diameter was measured using dynamic light scattering and AFM/TEM imaging.

#### Assembly of the AuNP-based nanostructure

To assemble the nanostructures (pairs of AuNPs or chains of three AuNPs), different types of DNA-conjugated AuNPs were mixed together in a 1 : 1 ratio, and incubated at room temperature for four hours. Structure verification was obtained using 1% agarose gel electrophoresis (8 V cm^−1^, 1 h, 0.5× TBE) and AFM/TEM imaging.

For the hybridization chain reaction, two hairpin probes were separately conjugated onto the DNA cage around the AuNPs, AuNP-H1 and AuNP-H2. Then, they were mixed together and incubated overnight at room temperature. An initiator strand was then added to trigger the reaction in a 1 : 3 ratio with the amount of each hairpin. After incubation for four hours, TEM imaging and 1% agarose gel electrophoresis were performed.

### AFM and TEM imaging

The assembled structures were deposited on freshly cleaned muscovite mica (Electron Microscopy Sciences) for five minutes, then rinsed with MQ-pure water and dried under nitrogen gas. AFM imaging was performed in air in ScanAsyst mode on a Veeco Multimode Scanning Probe Microscope with silicon Scanasyst-air probes (resonant frequency 50–90 kHz, spring constant 0.4 N m^−1^, and a tip radius of 2 nm).

For TEM imaging, a JEM 2010 transmission electron microscope (JEOL) operated at 200 kV was used. The images were analyzed using a GATAN MSC 794 CCD Camera and GATAN Digital Microscopy software. Five μL AuNP assemblies were transferred to SPI® Supplies Holey carbon coated grids onto 400 mesh copper, incubated for three minutes, washed with 50 μL of MQ-pure water, and dried under nitrogen gas. Samples were then dried completely for at least 15 hours under ambient conditions in a vacuum desiccator.

### Dynamic light scattering measurements

Measurements were performed using a ZetaPlus (Brookhaven Instruments Corporation). Incident light was provided by a 35 mW solid-state laser (660 nm). Scattered light was collected at a fixed angle of 90°. At least five runs were performed per sample, with 30 seconds per run.

## Results and discussion

### Assembly of the octahedron

To assemble a flexible structure that would be able to take up the gold nanoparticles from the solution, an octahedron model was chosen (Scheme 1). Octahedrons tend to be spherical in shape, but still have a simple multifaceted structure. DNA oligonucleotide sequences (Table S1†) were pre-designed using NUPACK^25^ to achieve a similar melting temperature between complementary regions and formed triangular faces. Each edge length was 23 bp (7.82 nm), which was long enough to occupy one quarter of a circle of the AuNPs used. The diameter of the nanoparticles was chosen to be 10 nm, so that it would match the size of the hairpins that will be used in the HCR cascade and allow the assembly of highly distinctive chains. To facilitate the bending of DNA strands at the corners of the cage, a single-stranded region of two thymines was added between the double-stranded edges. The octahedron cage was divided into two hemispheres, A and B, with four DNA strands each, which were preassembled in PBS buffer with 0.1 M NaCl and 5 mM MgCl_2_, and then stored at 4 °C until further usage without any additional purification from misassembled strands. The assemblies remained stable for up to two weeks in the described storage conditions. The quality of the stock was verified each time before the experiment by taking AFM images (Fig. S2†).

**Fig. 1:**
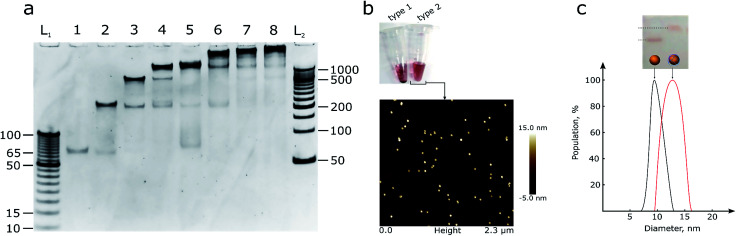
(a) 10% PAGE gel with DNA assemblies of octahedron parts. L_1_ and L_2_ – DNA ladders with 5 and 50 bp steps respectively, and the number of ssDNA oligonucleotides in the reaction mixture for octahedron assembly ranged from one to eight. (b) a photo of the two types of AuNPs, see text for details, and AFM image of type 2 AuNPs that does not show any aggregation signs. A picture with higher resolution can be seen in the ESI (Fig. S2).† (c) 2% agarose gel, from left to right: line 1 – control OEG-AuNPs, line 2 – OEG-AuNPs after mixing with the hemispheres and octahedron assembly; DLS measurement of the particle size: black – control AuNPs, red – AuNPs with octahedron assembly.

The quality of the obtained structure was verified using 10% PAGE (Fig. 1a): several reaction mixtures were prepared with a different number of ssDNA strands ranging from one to eight. Line 8 in Fig. 1a corresponds to the mixture with all the necessary DNA strands to form the desired product, while the rest act as controls containing different subsets of the reactant strands. The octahedron can be formed without any additional pre-assembly steps, and the size of the edges is big enough to facilitate the building of the preferred structure.

The hemispheres were mixed (Fig. 1b, S3†) with 10 nm AuNPs of two types: bare AuNPs (type 1) and thiol-OEG coated AuNPs (type 2) for stability control using a published dATP protocol.^12^ Type 1 AuNPs were not stable in buffers with a sodium concentration larger than 0.2 M and in the presence of Mg^2+^ ions, due to large areas that were not protected by DNA. So, although it was possible to create an octahedron assembly around bare AuNPs, thiol-OEG-protected AuNPs were used for further reactions in order to ensure robust DNA assembly and to reduce of the number of side products it is necessary to use bivalent ions. Moreover, the neutral surface of type 2 AuNPs did not interfere with the assembly process compared with bare, citrate-stabilized AuNPs. Type 2 AuNPs also did not show any signs of aggregation in solutions with high ionic strength. The neutral charge in type 2 AuNPs allowed us to use dsDNA to form polyhedrons with more faces than octahedrons and make assemblies in more stringent conditions. In addition to the AFM data, DLS analysis was performed (Fig. 1c) to control the size of the particles.

### Assembly of a complex of two and three AuNPs

**Scheme 2:**
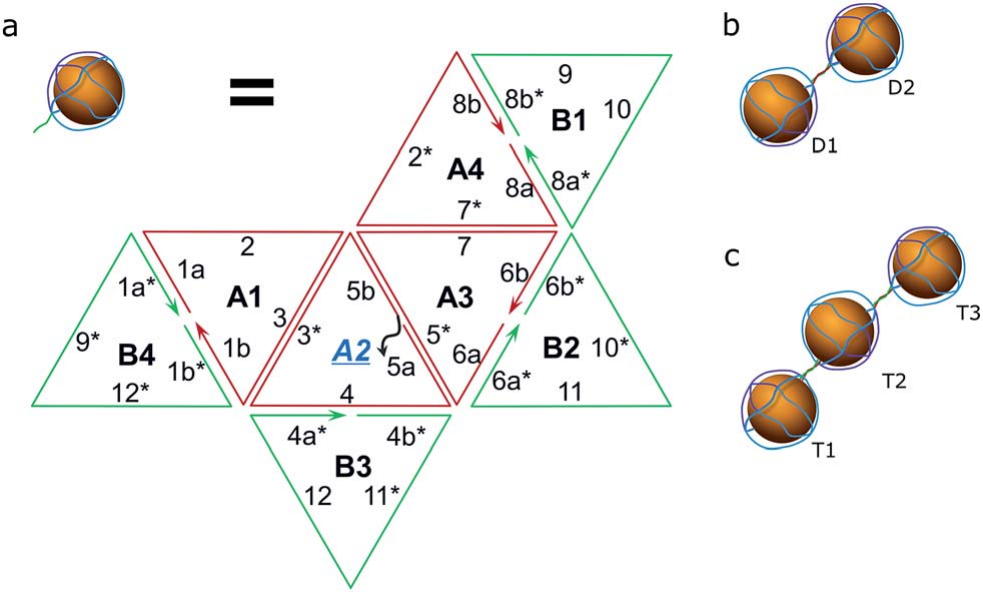
Hybridization scheme. (a) The composition of the octahedron (two hemispheres – green and red) with sticky toeholds (grey) for type D1, D2, T1 or T3 of the AuNPs; note: type 2 AuNPs have toeholds in positions A2 and A4, see S2.† (b) The resulting pair of AuNPs. (c) A trio of AuNPs.

**Fig. 2:**
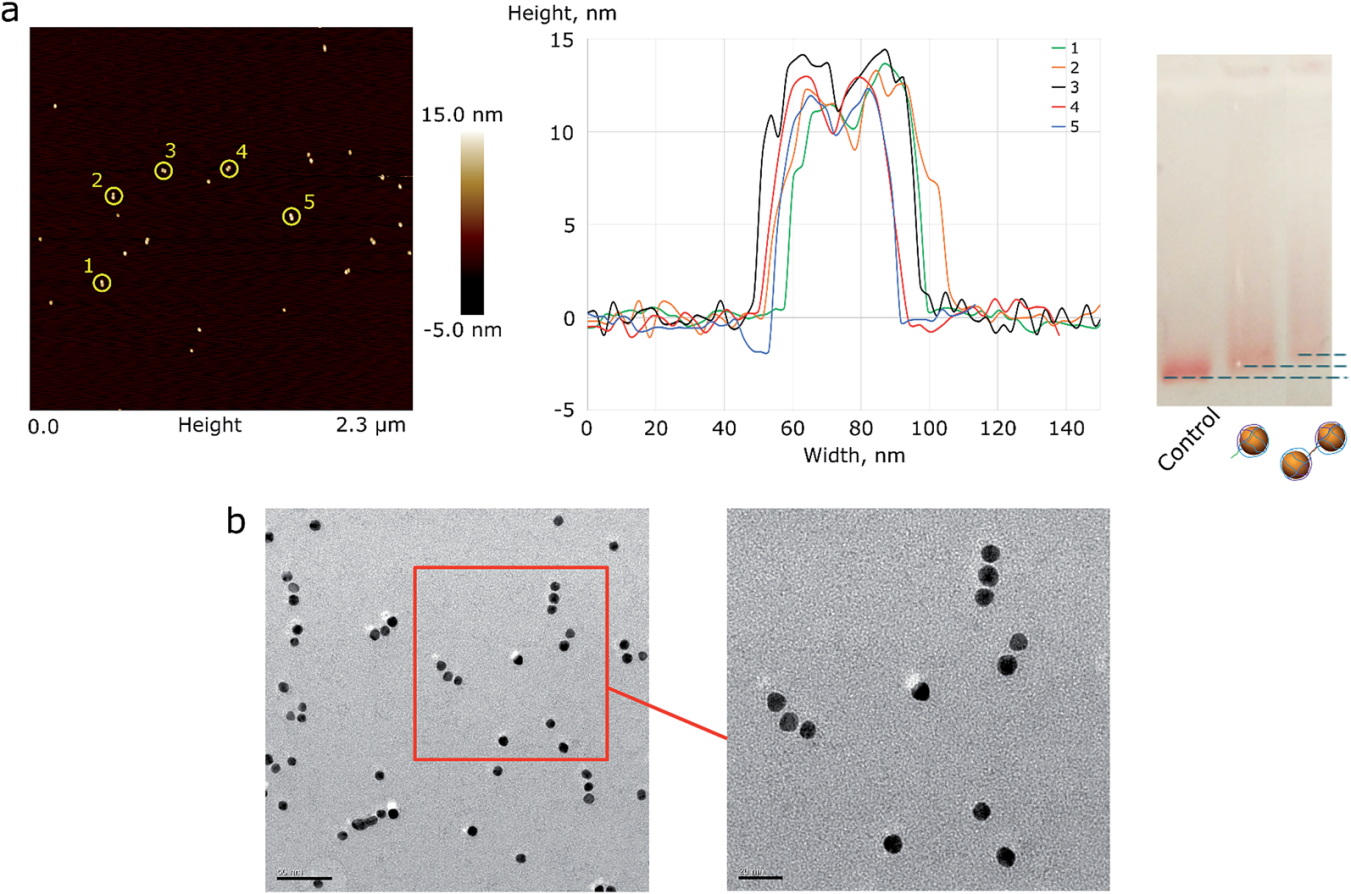
(a) AFM image of AuNP pair assemblies with height profile and a 1% AGE control. A picture with higher resolution can be seen in the ESI (Fig. S4†). (b) The TEM image of triple AuNP assemblies; the scale bar corresponds to 50 nm (left) and 20 nm (right).

As a proof of concept, the assembly of two and three AuNPs was chosen. The DNA structure for dual (AuNP types D1 and D2) and triple AuNP (types T1, T2, and T3) assemblies had additional single-stranded DNA toeholds, complementary to each other (*e.g.*, the toeholds of the D1 structure hybridized to D2 toeholds – for a further explanation see Schemes 2 and S4†). The toehold length was 10 nt, corresponding to a 3.4 nm theoretical distance between nanoparticles. Pre-assembled AuNP complexes of different types were mixed in equimolar concentrations and then incubated overnight at room temperature. The quality of the assembly was verified using AFM/TEM imaging and gel electrophoresis (Fig. 2a and S5†). The T2 type of AuNPs has 2 toeholds, with a 180 degree angle between them. The unified chain-like shape of triple assemblies with equal distances between neighbouring nanoparticles (Fig. 2b) clearly shows that it is possible to control the placement of DNA sticky ends for further nanoscale construction.

Based on the TEM image analysis, the yield of triple assemblies for type 1 AuNPs was 40%. This yield was the consequence of the reaction conditions: low salt concentrations were used in order to prevent the random aggregation of nanoparticles, because only ∼34% of the nanoparticle's surface was protected by DNA. When type 2 AuNPs were used, the yield was nearly 70%. The yield was measured based on the band intensity in agarose gel electrophoresis.

The yield percentage may be further controlled by varying the toehold length. A long toehold makes hybridization more accessible, and the melting temperature for the assembly is higher, which increases the number of successful constructions.

### Assembly of AuNP chains

**Fig. 3:**
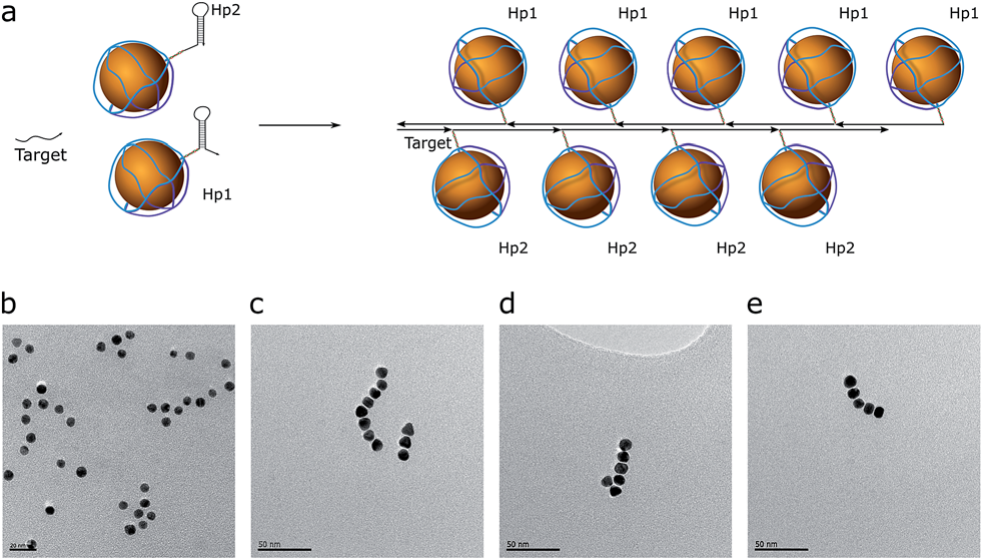
Chain assembly by the HCR reaction. (a) Assembly scheme. Hairpins (Hp1 and Hp2) are drawn in a proportional size to the nanoparticles. (b) The TEM image of the control mixture. (c–e) TEM images of obtained assemblies.

The hybridization chain reaction (HCR) was used for chain assembly. This is a cascade reaction, where the two AuNP-hairpins are opened by a trigger strand (target) and form a polymeric double-stranded product (Fig. 3a).^27^ These hairpins are kinetically trapped (Fig. 3b), *i.e.*, they are stable in solution without a target (initiator) strand, which enables us to monitor the product, which is a concatenated polymer of dsDNA. The hairpins have an input domain in the exposed ssDNA region (toehold) and an output domain in the loop. The target hybridizes with the toehold and opens the loop of one of the hairpins. The exposed loop can then hybridize with a toehold of the second hairpin, which has a sequence identical to the target; thus, the cascade repeats over and over again, leading to a polymerized product (Fig. 3c–e). The design process was done at the domain level. A DNA Strand Displacement tool (vDSD)^28–30^ was used to predict possible side reactions and to build computational models. Sequence design was made using NUPACK^26^ software in such a way that all intermediate products, hairpins, and reaction yields have a negative Δ*G*^0^.

Based on the control reaction without AuNPs (Fig. S6†), the estimated length of the product should correspond to a chain of 20–30 AuNPs. However, the maximum length that could be achieved was 8–10 AuNPs. This could be explained by the assumption that HCR product polymerization goes through a chain-growth mechanism with a very fast initiation rate and a slow propagation rate. Also, the distribution of chain length follows a Poisson distribution shape, meaning that the most probable product length to be observed in this condition is 5–7 AuNPs per chain.

### Estimation of possible chain length

The product of the HCR could be defined as a polymer chain, where the hairpins play the role of monomers. There are several hypotheses to characterize the polymerization mechanism: step-growth and chain-growth with its subform of living polymerization. The HCR is an example of chain-growth polymerization due to the calculation that its initiation rate is higher than its propagation rate (*k*_init_ = 8.7 × 10^6^ M^−1^ s^−1^ and *k*_prop_ = 9.0 × 10^5^ M^−1^ s^−1^; rate constants were calculated according to the procedure described by J. X. Zhang *et al.*^31^). Theoretically, the reaction does not stop until all the monomers are consumed, so it can be called a class of living polymerization.

**Fig. 4:**
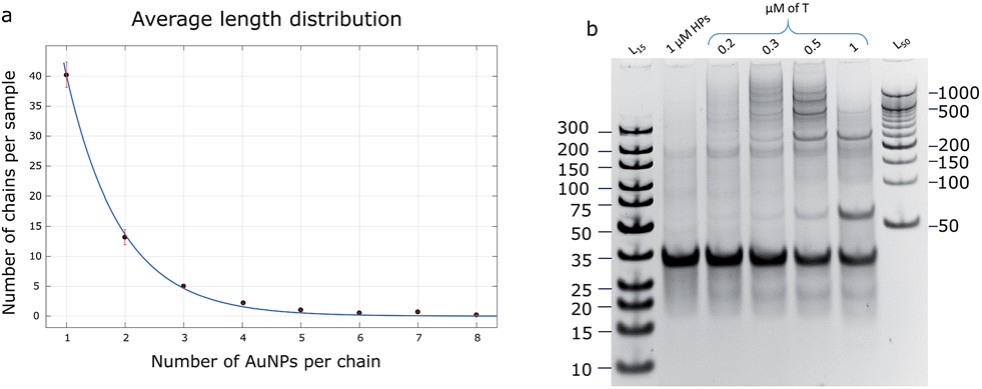
(a) Average length distribution of the HCR product with AuNPs. The trendline is shown in blue and error bars are in red. (b) A 10% PAGE gel with DNA HCR products: lanes 1 and 7 – DNA ladder, lane 2 – control (Hp1 and Hp2 only, no target), lanes 3, 4, 5, and 6 – HCR reaction with different target concentrations. To obtain longer DNA chains, the ratio of HPs : target should be larger than 1.

The average length distribution of HCR chains was calculated based on the obtained TEM images (40 000× magnification) for AuNP assemblies (Fig. 4a and S7†) and PAGE gels for DNA controls. All calculations and curve-fitting processes were performed using Matlab. The degree of polymerization fits a Poisson distribution, which proves our assumption that the HCR follows a chain-growth mechanism.^32,33^ According to the DNA control lane in the PAGE gel (Fig. 4b), the longest chains should be equal to or longer than 10 monomers; however, the TEM image shows that the longest observed chain is eight AuNPs, which may be due to the fact that when the chain grows, the particle concentration decreases, and the possibility of a collision between the monomers and growing polymers drops too, so the probability of observing a long chain in the TEM image is low.

## Conclusions

The proposed protocol can be further used for nanoscale construction. A DNA cage can be designed to contain more edges for more complex assemblies. The advantage of this method is the simplicity of its design – there is no need to use additional programs or computing power. Nanoparticles with diameters equal to 10 nm were used in this work; however, for larger AuNPs, the elongation of DNA strands may be required due to the limitations of DNA synthesis protocols. Future efforts should focus on work with cascades of DNA displacement reactions for electrochemical devices, nucleic acid detection and its delivery into living cells, and the possibility of obtaining structures of a higher order.

## Conflicts of interest

There are no conflicts to declare.

## Supplementary Material

NA-001-C8NA00148K-s001
