# Supplementary material for: Thiol-free oligonucleotide surface modification of gold nanoparticles for nanostructure assembly
Source: Nanoscale Adv. 2018 Sep 20;1(1):430–5. doi: 10.1039/c8na00148k (PMC9473237; doi:10.1039/c8na00148k)
Supplement: NA-001-C8NA00148K-s001 [file NA-001-C8NA00148K-s001.pdf]

Electronic Supplementary Material (ESI) for Nanoscale Advances.
This journal is © The Royal Society of Chemistry 2018

Supporting Information

# Thiol-free oligonucleotide surface modification of gold nanoparticles for nanostructure assembly

Anastasia Maslova,[a] I-Ming Hsing*[a]

## S1 – DNA sequences

| Name | Sequence |
|---|---|
| A1 | ATGACCATGTTATTACGAATTCGAGCTCGGTATTCCCGGGGATCCTCTAGAGTCGTTAGCTG<br>CAGCCAT |
| A2 | CGTATGTTGATCGACTCTAGAGGATCCCCGGGTTGCACTGGCCGTCGTGG<br>TACAATTCGTCGTGACTGG |
| A2-l | CGTATGTTGATCGACTCTAGAGGATCCCCGGGTTGCACTGGCCGTCGTGGTACAATTCGTCG<br>TGACTGGCTTTCGTCCT |
| A2-llc | CGTATGTTGATCGACTCTAGAGGATCCCCGGGTTGCACTGGCCGTCGTGGTACAATTCGTCG<br>TGACTGGAGGACGAAAG |
| A3 | TCCTGACGTTTTTGTACCACGACGGCCAGTGCTTACCCAACTTAATCGCCTTGCATTGCACA<br>TCCTTCG |
| A4 | CCAGCTGCCTTTGCAAGGCGATTAAGTTGGGTTTTACCGAGCTCGAATTCGTAATTTGTAAT<br>AGCGAAG |
| A4-I | CGAATTCGTAATTTGTAATAGCGAAGCCAGCTGGCTTTGCAAGGCGATTAAGTTGGGTTTTA<br>CCGAGCTCTTTCGTCCT |
| A4-IIc | CGAATTCGTAATTTGTAATAGCGAAGCCAGCTGGCTTTGCAAGGCGATTAAGTTGGGTTTTA<br>CCGAGCTAGGACGAAAG |
| B1 | CTTCGCTATTACTTTACGAGTTGTCGAATTGTTTGTTTAAAGTCTAATACTTCTACCTTTGG<br>CAGCTGG |
| B2 | CGAAGGATGTGCTTAGGTAGAAGTATTAGACTTTATTCTTCGCTATTACGCCAGCTGGTTAC<br>GACAGGA |
| B3 | CCAGTCACGACGTTCCAGCTGGCGTAATAGCGAAGTTCCTCAAATGTATTATCTATTGTTCA<br>ACATACG |
| B4 | ATGCGTGCAGCTTTCAATAGATAATACATTTGAGGTTCAAACAATTCGACAACTCGTATTCA<br>TGGTCAT |

Table S1. DNA sequences used for octahedron assembly.

Comment: ‘ or * sign shows complementarity (e.g. 11* region is complementary to 11 region), letters a and b correspond to 3’ and 5’ ends of DNA strands (see scheme below). Name xx-I and xx-IIc shows complementary

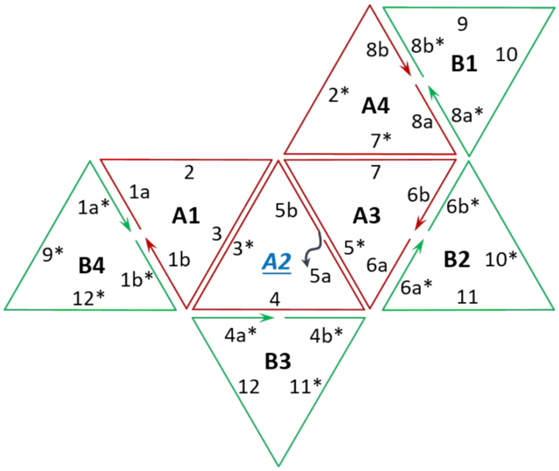


Figure S1. Default octahedron assembly with one ssDNA toehold.

toeholds for equal strands (for example, A2-I belongs to scaffold type 1 and hybridizes with A2-IIc of scaffold D2).

## S2 – AFM image of control stock of encapsulated AuNPs in DNA cage.

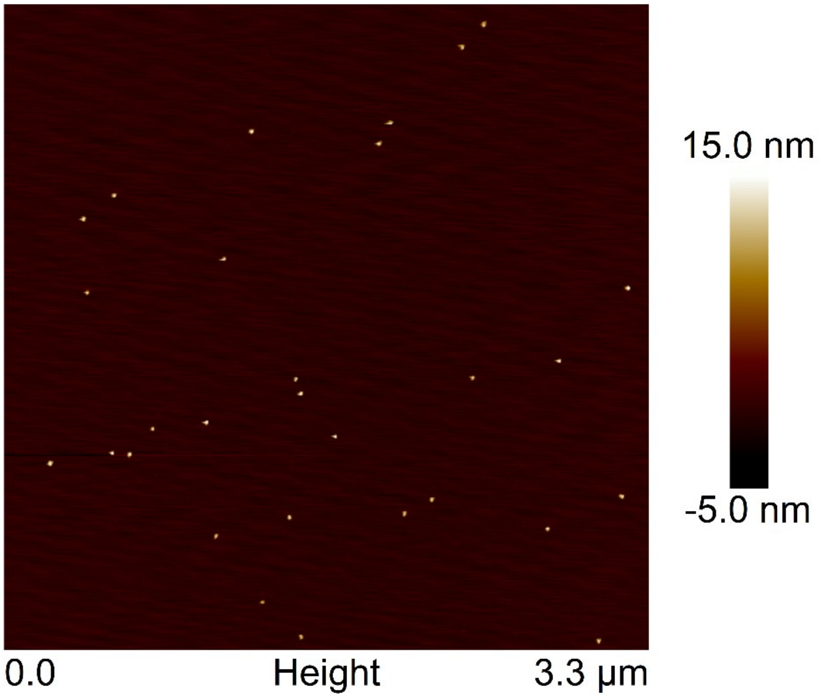

S3 – AFM image of type 2 AuNPs

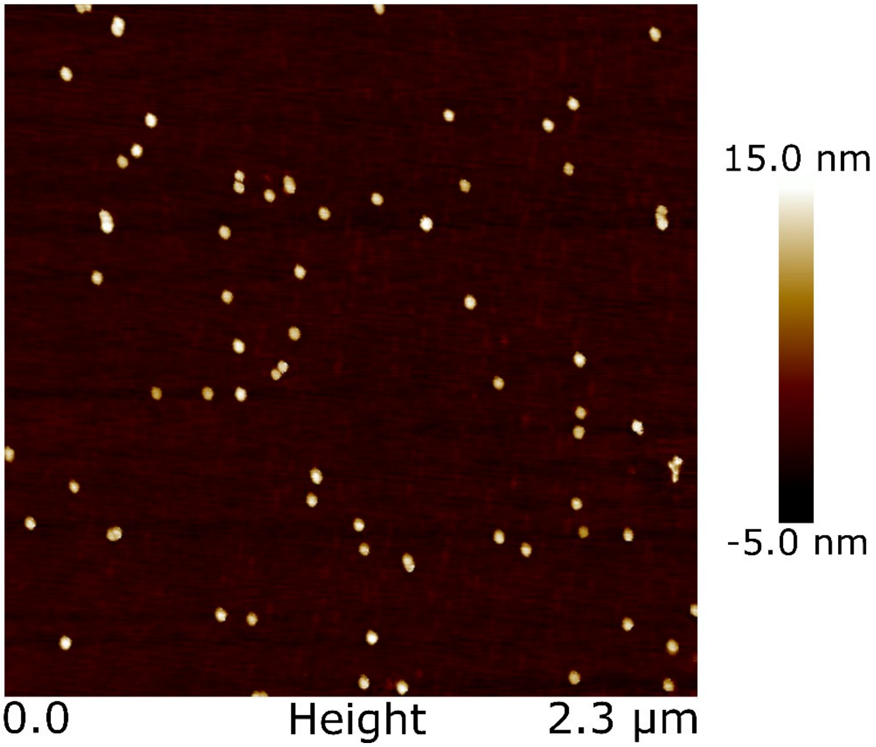


Figure S3. AFM image of type 2 AuNPs

## S4 – Principal scheme of T2 type of AuNPs

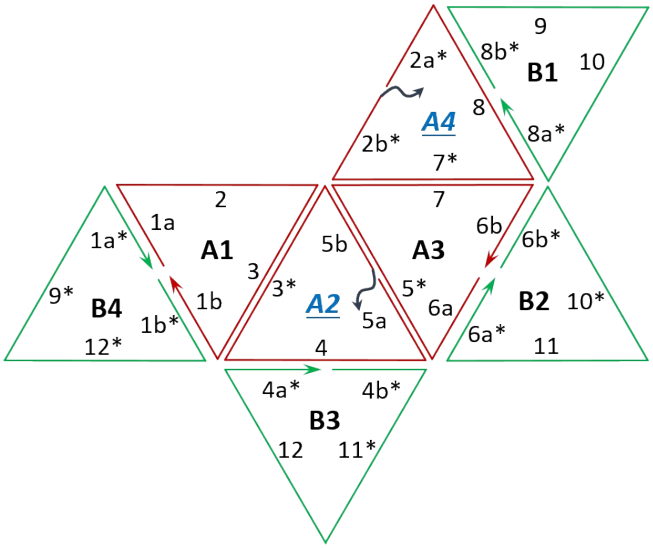


Figure S4. T2 type octahedron.

## S5 – AFM image of AuNPs pairs assembly

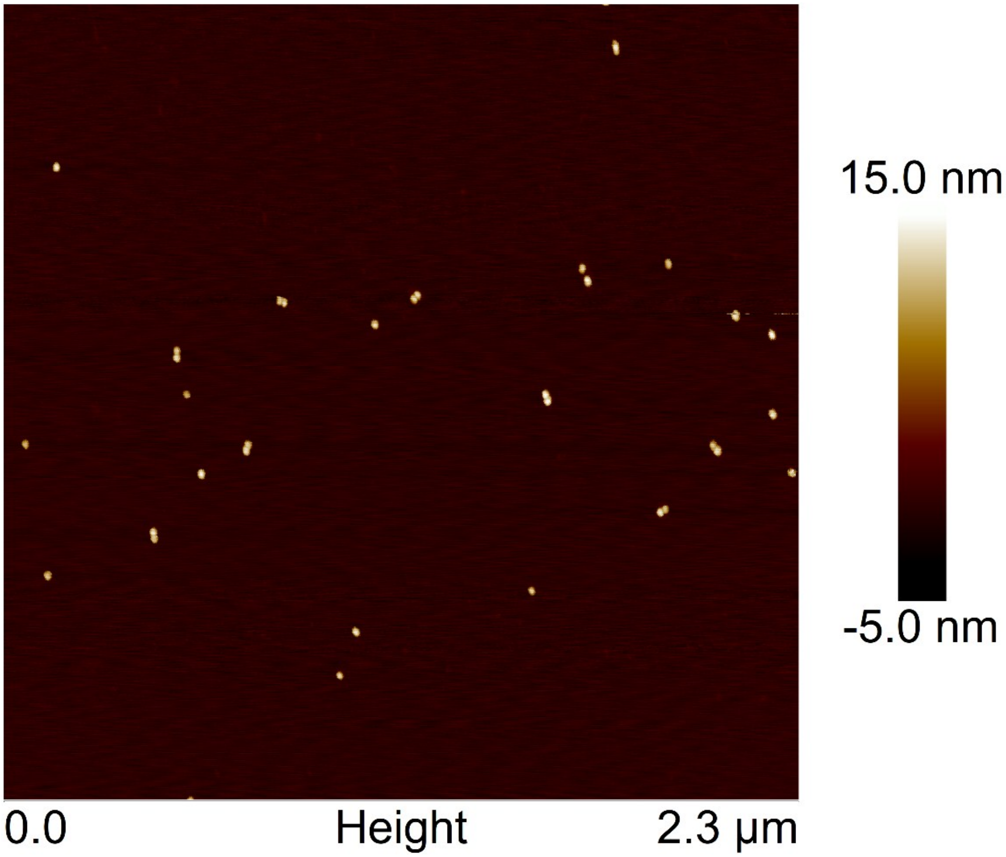


Figure S5. AFM image of AuNPs pairs assembly.

## S6 – HCR reaction

| Name | Sequence |
|---|---|
| Target | GCAGGAGTAGAAGATGGAGCAGC |
| Hp 1 | AAAAAAAAAAGCTGCTCCATCTTCTACTCCTGCATCCGGGCAGGAGTAGA AGATGG |
| Hp2 | AAAAAAAAAAGCAGGAGTAGAAGATGGAGCAGCCCATCTTCTACTCCTGCCCGGAT |

DNA sequences for hairpins and a target:

Table S6. DNA sequences for HCR reaction: Hp1 and Hp2 stand for hairpin 1 and hairpin 2.

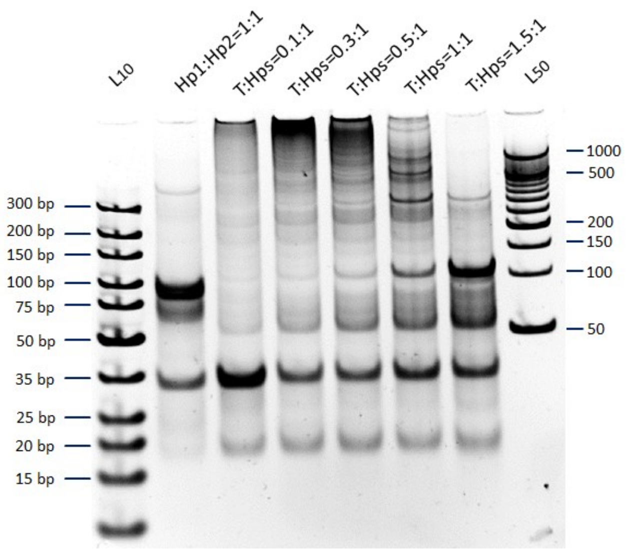


Figure S6. Lane 2: Control mixture of 2 hairpins without a target. Lanes 3-7: reaction mixture of 2 hairpins and the target in different molar ratio.

10% PAGE gel for HCR products (without AuNPs):

## S7 – Polymerization

TEM images were taken of six samples with HCR reaction products. Number of chains were calculated (Table S3, Fig. S4):

| Length (AuNPs) | Sample 1 | Sample 2 | Sample 3 | Sample 4 | Sample 5 | Sample 6 | Average length | St. error |
|---|---|---|---|---|---|---|---|---|
| 1 | 50 | 38 | 40 | 32 | 56 | 25 | 40.16667 | 4.245368 |
| 2 | 27 | 12 | 11 | 12 | 9 | 8 | 13.16667 | 2.597185 |
| 3 | 4 | 3 | 9 | 5 | 4 | 5 | 5 | 0.781736 |
| 4 | 0 | 3 | 2 | 3 | 2 | 2 | 2 | 0.408248 |
| 5 | 2 | 1 | 1 | 0 | 2 | 0 | 1 | 0.333333 |
| 6 | 0 | 0 | 2 | 0 | 1 | 0 | 0.5 | 0.311805 |
| 7 | 1 | 1 | 0 | 2 | 0 | 0 | 0.666667 | 0.30429 |

| | | | | | | | | |
|---|---|---|---|---|---|---|---|---|
| 8 | 0 | 0 | 1 | 0 | 0 | 0 | 0.166667 | 0.152145 |

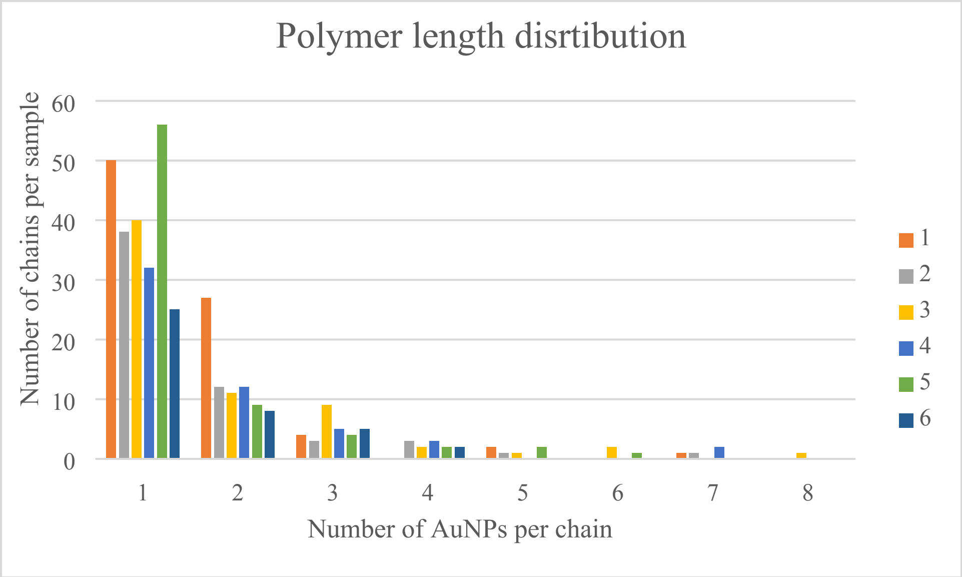


Figure S7. Chains length distribution based on Table S7.

Table S7. Number of chains with designated length in different samples.
